# The Association of Cardiorespiratory Fitness on Memory Function: Systematic Review

**DOI:** 10.3390/medicina55050127

**Published:** 2019-05-09

**Authors:** Brandon Rigdon, Paul D. Loprinzi

**Affiliations:** Exercise & Memory Laboratory, Department of Health, Exercise Science and Recreation Management, The University of Mississippi, University, MS 38677, USA; btrigdon@go.olemiss.edu

**Keywords:** cognition, cognitive function, episodic memory, physical activity, working memory

## Abstract

*Background and Objectives*: Cardiorespiratory fitness is an important predictor of cardiovascular and cardiometabolic health. To extend our knowledge on the health effects associated with cardiorespiratory fitness, the objective of this study was to evaluate the association of cardiorespiratory fitness on memory function. *Materials and Methods:* Embase/PubMed, Web of Science, Google Scholar, Sports Discus, and PsychInfo databases were searched. Inclusionary criteria included: (1) were conducted among adult humans (18+ years), (2) evaluated cardiorespiratory fitness as the independent variable, (3) measured cardiorespiratory fitness with an objective device (e.g., indirect calorimetry), (4) evaluated memory function (any type) as the outcome measure, and (5) included either a cross-sectional, prospective, or experimental-study design. Information on the participant’s characteristics, study design, cardiorespiratory fitness assessment, memory type, whether the study statistically controlled for exercise behavior, and study results were extracted. The relationship between cardiorespiratory fitness and memory was synthesized while considering the data extraction parameters. *Results:* In total, 17 articles met the inclusionary criteria, including two prospective cohort studies and 15 cross-sectional studies. The main findings of this review are twofold: (1) across the 17 evaluated studies, 15 (88.2%) studies demonstrated some evidence of a positive association between cardiorespiratory fitness (CRF) and memory function, and (2) none of these 17 studies statistically controlled for physical activity behavior. *Conclusion:* CRF appears to be positively associated with memory function, however, it is uncertain as to whether this association occurs independently of physical activity or is mediated via physical activity behavior.

## 1. Introduction

Unquestionably, memory function is critical for daily functioning. Memory is a complex neuropsychological system, involving several memory subsystems, including various processes and streams of information subserving their respective systems. Working memory capacity, a short-term memory system, involves the transient storage of information with concomitant interfering stimuli. The declarative long-term memory system involves explicit and implicit sub-streams with the former involving conscious encoding of material and the latter involving subconscious encoding of information. Further, within the explicit declarative memory system, episodic memory refers to retrospective memories that are bound contextually whereas semantic memory involves non-contextually bound retrospective memories [1,2,3].

Of interest to our research group are the effects of exercise on episodic memory function. In various reviews [1,4] and empirical experiments [5,6,7,8], we have provided suggestive evidence that both acute and chronic engagement in exercise may subserve various memory systems, particularly episodic memory function. The focus of this review is to evaluate the potential unique effects (i.e., independent of exercise) of cardiorespiratory fitness (CRF) on memory function.

Cardiorespiratory fitness is defined as the ability of the circulatory and respiratory systems to transport and supply oxygen to skeletal muscles. As determined from the Fick equation, CRF is influenced by central and peripheral factors. Specifically, CRF is a function of cardiac output (central) and arteriovenous difference (peripheral). Enhanced transport of oxygenated blood and enhanced extraction of oxygen at the muscle cell level, corresponds with higher CRF.

Emerging research demonstrates that, independently of exercise, CRF is associated with various health-related outcomes [9] including mortality risk [10,11]. We have also demonstrated that, independently of exercise and sedentary behavior, CRF is positively associated with cognitive function [12]. Importantly, however, objective measures of CRF should be considered as our recent work demonstrates that estimates of CRF in young adults are not associated with memory function [13].

Future work on this topic is needed, and as such, the purpose of this paper was to systematically review the literature to evaluate the extent to which objectively-determined CRF is associated with memory function. A particular interest of this review was to evaluate whether this potential relationship is independent of exercise engagement. Such an effect is plausible as CRF and exercise may include distinct components of cardiovascular health [14]. As an example, the association between exercise engagement and CRF is weak-to-modest at best (r = 0.30–0.60) [15,16]. Further, CRF may not only be influenced by exercise engagement, but may also be influenced by subclinical disease and genetic predisposition [17].

## 2. Methods

PRISMA guidelines were utilized as a framework for this systematic review.

### 2.1. Data Sources and Search Strategy

The following databases were used for our computerized searches: Embase/PubMed, Web of Science, Google Scholar, Sports Discus, and PsychInfo [18]. Articles were retrieved from inception to 14 March 2019. The search terms including their combinations were: fitness, maximum oxygen consumption, aerobic fitness, aerobic capacity, cardiorespiratory fitness, cognition, memory, and episodic memory. Each independent variable (e.g., cardiorespiratory fitness, aerobic fitness) was searched with each outcome variable (cognition, episodic memory). An example search strategy is shown below:

(“cardiorespiratory fitness” [MeSH Terms] OR (“cardiorespiratory” [All Fields] AND “fitness” [All Fields]) OR “cardiorespiratory fitness” [All Fields]) AND (“memory” [MeSH Terms] OR “memory” [All Fields])

### 2.2. Study Selection

The literature searches were performed independently by two separate authors, and comparisons were made to determine the number of eligible studies. Consensus was reached from these two independent reviews. Upon performing the computerized searches, the article titles and abstracts were reviewed to identify potentially relevant articles. Articles appearing to meet the inclusionary criteria were retrieved and reviewed at the full text level.

### 2.3. Inclusionary Criteria

Studies were included if they: (1) were conducted among adult humans (18+ years), (2) evaluated cardiorespiratory fitness as the independent variable, (3) measured cardiorespiratory fitness with an objective device (e.g., indirect calorimetry), (4) evaluated memory function (any type) as the outcome measure, and (5) included either a cross-sectional, prospective, or experimental study design.

### 2.4. Data Extraction of Included Studies

Detailed information from each of the included studies were extracted, including the following information: author, study design, population characteristics, cardiorespiratory fitness assessment, memory type, whether the study statistically controlled for physical activity behavior, and study results. Notably, a qualitative—systematic—review was employed as opposed to a quantitative—meta-analysis—review because of study design heterogeneity, population heterogeneity, and variability in the reported outcome metrics (e.g., F-values, correlation coefficients, beta coefficients, relative risk estimates).

## 3. Results

### 3.1. Retrieved Articles

**Figure 1:**
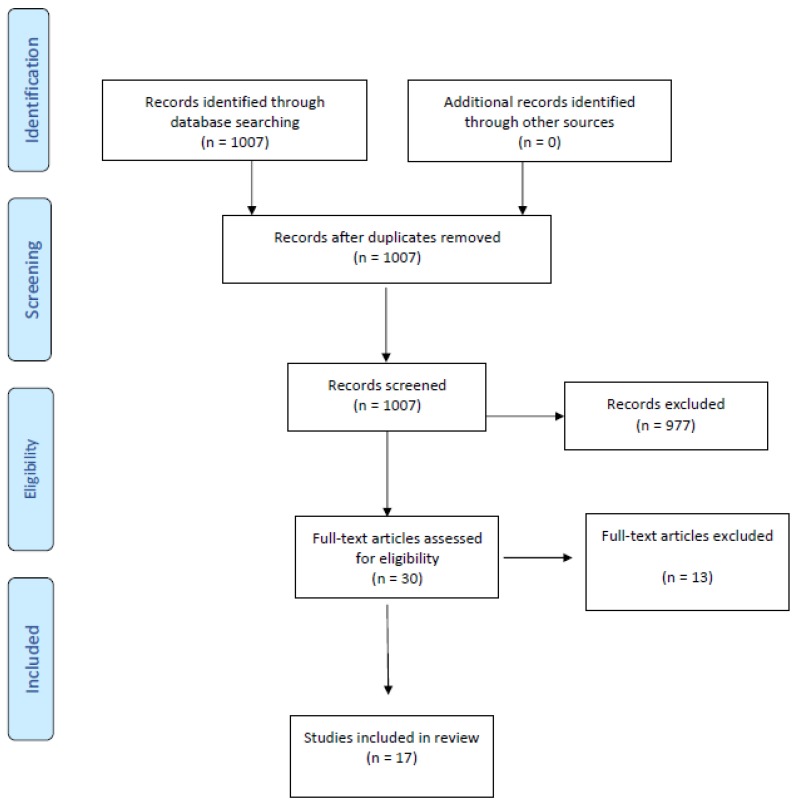
Flow chart of article retrieval.

Figure 1 displays the flow chart of the article retrieval process. The computerized searches identified 1007 articles. Among the 1007 articles, 977 were excluded and 30 full-text articles were reviewed. Among these 30 articles, 13 were ineligible as they did not meet our study criteria. Thus, in total, 17 articles met our inclusionary criteria and were evaluated herein.

### 3.2. Article Synthesis

Details of the study’s characteristics are displayed in Table 1 (extraction table). As shown in table 1, 17 studies evaluated the association between CRF and memory. Of these 17, there were two prospective cohort studies (six-year follow-up and 18-year follow-up periods) and 15 cross-sectional studies. Among the 17 studies, only five focused on young adults (<35 years) [19,20,21,22,23], with the remaining studies focused on middle-age or older adults.

All 17 studies employed an objective measure of cardiorespiratory fitness, which involved a maximal exercise test (as opposed to VO_2max_ estimated from a submaximal test). Among the 17 studies, various memory systems were evaluated, including working memory, spatial memory, episodic memory, and source memory.

Among the 17 studies, none statistically controlled for physical activity when evaluating the relationship between CRF and memory function. Among the 17 studies, 12 did not mention anything about physical activity level being an inclusionary/exclusionary criterion, and thus, the level of physical activity behavior among the majority of these studies (12/17; 70.6%) is uncertain. However, one of these studies (5.9%) sampled both sedentary and endurance athletes at baseline, and four of the 17 studies (23.5%) only sampled relatively inactive individuals. Regarding these four latter studies, however, the criteria for being inactive varied, including being “inactive”, “low active”, “not very physically active” (less than two days per week of physical activity in the past six months), and “inactive” (less than 30 min per week of physical activity within the past six months). Thus, even among these four studies that, by study design, only included “inactive” individuals, it is likely that there was considerable variability in their physical activity levels (not only at moderate-to-vigorous physical activity intensity, but also at light-intensity physical activity).

Among the two prospective cohort studies [24,25], one study employed a six-year follow-up period among older adults and showed that baseline CRF was positively and prospectively associated with verbal memory and verbal fluency (*p* < 0.01). The other prospective cohort [25], which employed an 18-year follow-up period among adults 19-94 years, showed that lower levels of CRF were associated with a greater decline in learning and memory function.

Among the 15 cross-sectional studies [19,20,21,22,23,26,27,28,29,30,31,32,33,34,35], 13 demonstrated a statistically significant positive association between CRF and memory function, whereas two studies [30,32] demonstrated no statistically significant association between CRF and memory. Thus, the majority of these cross-sectional studies (86.7%; 13/15) demonstrated a positive relationship between CRF and memory. Importantly, however, among the 13 studies demonstrating a positive association between CRF and memory, not all of the analyses within each study demonstrated a positive association. For example, Oberlin et al. (2015) [31] demonstrated a positive association between CRF and memory in their second cohort (β = 0.237, *p* = 0.006) but not in their first cohort (β = 0.17, *p* = 0.15). Tarumi et al. (2015) [29] showed that although CRF (when expressed in mL/kg/min) was not associated with memory, when expressed as a percentile, it was statistically significantly associated with memory. Hayes et al. (2016) [21] demonstrated that CRF was not associated with memory in young adults but was statistically significantly positively associated with memory in older adults (F = 5.40, *p* < 0.05). Further, when examined across the entire sample, Dougherty et al. (2017) [28] demonstrated no overall statistically significant association between CRF and memory, but CRF was positively and statistically significantly associated with memory among men. Lastly, Brown et al. (2019) [26] did not observe an association between CRF and verbal memory but did observe a statistically significant positive association between CRF and paired associative memory.

## 4. Discussion

The emerging field of exercise neurophysiology has accumulated evidence suggesting that acute and chronic exercise may subserve memory function. Less work, however, has focused on the effects of CRF on memory function. Further, it is uncertain as to whether the potential relationship between CRF and memory is independent of exercise behavior or is mediated by exercise levels. As a result, the purpose of this review was to evaluate the literature to determine the association between CRF and memory and evaluate the extent to which exercise has been considered in this relationship. The main findings of this review are twofold: (1) across the 17 evaluated studies, 15 studies (88.2%) demonstrated some evidence of a positive association between CRF and memory function, and (2) none of these 17 studies statistically controlled for exercise behavior.

Previous work suggests that CRF and exercise may have distinct effects on health [14]. This stems from several observations. First, exercise behavior and CRF are only modestly correlated [15,16]. Second, CRF has been shown to be a stronger predictor of cardiovascular health when compared to exercise behavior [14]. Thirdly, approximately 40–50% of an individual’s CRF has been suggested to be influenced by their genetic profile [36]. Collectively, this suggests that both CRF and exercise may play distinct roles in cardiovascular health, and by extension, cognitive health [37]. However, this needs to be carefully and critically evaluated. The modest correlation between CRF and exercise may, in part, be influenced by measurement error associated with the assessment of exercise behavior. This may also explain the relatively stronger associations between CRF (vs exercise) and cardiovascular health. Lastly, considerable work still needs to be conducted to determine the extent to which specific genes and combinations of genetic factors contribute to inter-individual variability in CRF and CRF adaptations to exercise training interventions [38].

Strengths of this review include the novelty of this paper, the comprehensive approach taken, and evaluating whether physical activity was considered in the CRF-memory relationship. A limitation, however, is that we were unable to quantitatively evaluate the results, as a meta-analysis was not appropriate given the considerable heterogeneity across multiple study parameters (e.g., study design). Future work that carefully evaluates the potential independent and/or synergistic effects of CRF and exercise on memory is needed. If such work demonstrates a unique role of CRF on memory, then candidate mechanisms will need to be identified. Relatedly, we recently demonstrated that higher CRF was associated with greater interhemispheric parahippocampal connectivity [39], which likely plays an important role in subserving episodic memory function. Future work should also evaluate whether CRF moderates the intensity-specific effects of acute exercise on memory function [40].

## 5. Conclusions

In conclusion, the present review demonstrates a consistent association between CRF and memory function, occurring across various populations and memory systems. Thus, efforts to maximize and preserve CRF during aging should be a high public health priority.

## Figures and Tables

**Table 1:** Extraction table of the evaluated studies.

Author	Design	Population	Cardiorespiratory Fitness Assessment	Memory Type	Excluded Active Individuals at Baseline?	Statically Controlled for PA?	Results
Barnes et al. (2003) [24]	6-year prospective cohort study	*n* = 349 older adults (59–88 years); M_age_ = 69 years; 97% white	Indirect calorimetry; max test	Verbal memory and verbal fluency	Not mentioned	No	Baseline CRF was positively and prospectively associated with verbal memory and verbal fluency (*p* < 0.01)
Burns et al. (2008) [35]	Cross-sectional	*n* = 64 non-demented; *n* = 57 Alzheimer’s Disease; 52.3% female non-demented; 47.7% female with Alzheimer’s Disease	Indirect calorimetry; max test	Working memory; logical memory	Not mentioned	No	In early AD, CRF was positively associated with delayed memory (r = 0.30, *p* = 0.02) and digit symbol memory (r = 0.37, *p* = 0.05). In non-demented controls, CRF was positively associated with delayed memory (r = 0.26, *p* = 0.04)
Voss et al. (2010) [19]	Cross-sectional	*n* = 32 young adult; M_age_ = 24.1 years; 85% female; *n* = 120 elderly participants; M_age_ = 66.5 y; 71% female	Indirect calorimetry; max test	Spatial memory and working memory	Not mentioned	No	CRF was associated with fewer perseverative errors on the working memory task, *r* = −0.19, *p* <0.05. CRF was associated with better average accuracy and mean response time across all levels of the spatial memory task (Accuracy: *r* = 0.33, *p* <0.05; RT: *r* = −.31, *p* <0.05)
Szabo et al. (2011) [34]	Cross-sectional study	*n* = 158 older adults; M_age_ = 66.49 years; 65.4 % female; VO_2max_ = 20.92 mL/kg	Indirect calorimetry; max test	Spatial working memory	Yes, excluded “active” individuals	No	CRF was inversely associated with working memory reaction time (*r =* –0.33) and positively associated with working memory accuracy (*r =* 0.309)
Weinstein et al. (2011) [33]	Cross-sectional	*n* = 139; M_age_ = 66.6 years	Indirect calorimetry; max test	Spatial working memory	Yes, only included “low active” individuals	No	CRF was positively associated with greater working memory performance (F = 13.91, *p* < 0.01)
Erickson et al. (2012) [32]	Cross-sectional	*n* = 137; M_age_ = 66.08 years; 65.7% female; V0_2max_ = 21.32 mL/kg/min	Indirect calorimetry; max test	Digit span task and spatial memory task	Yes, only included individuals “not very physically active” as defined by participation in physical activity on two or fewer days of the week in the past six months	No	Aerobic fitness levels were not correlated with backward digit span lengths (*r* = 0.107; *p* = 0.23); or with forward digit span lengths (*r* = 0.124; *p* = 0.16)
Wendell et al. (2013) [25]	Prospective cohort, with follow-up to 18-years	*n* = 14,000 ages 19–94; 115 participants died and 46 withdrew; VO_2max_ = 28.6 mL O_2_/kg/min	Indirect calorimetry; max test	Visual and verbal memory	Not mentioned	No	Lower levels of CRF were associated with a greater decline in learning and memory function
Oberlin et al. (2015) [31]	Cross-sectional	Study 1, *n* = 113; 36.3% male Study 2, *n* = 154; 31.20% male; M_age_ = 66.6 years	Indirect calorimetry; max test	Spatial working memory	Yes, only included “physically inactive individuals” defined by engaging in 30 min or less each week of physical activity within the past 6 months	No	CRF was not associated with spatial working memory (β = 0.17, *p* = 0.15) for Experiment 1, but was positively associated with spatial working memory for Study 2 (β = 0.237, *p* = 0.006)
Schultz et al. (2015) [30]	Cross-sectional	*n* = 69; late middle-aged adults between the ages of 40–65; VO_2peak_ was 25.95 ± 5.50 mL/kg/min	Indirect calorimetry; max test	Working memory, immediate memory, verbal and learning memory	Not mentioned	No	No significant main effect associations between CRF and memory
Tarumi et al. (2015) [29]	Cross-sectional	*n* = 55 community dwelling adults, aged 43–65; M_age_ = 54 y for sedentary; M_age_ = 52 years for endurance trained; VO_2max_ = 26 ml/min/kg sedentary; VO_2max_ = 43 mL/kg/min	Indirect calorimetry; max test	Episodic memory (CAVLT)	Evaluated both sedentary and endurance athletes	No	Aerobic fitness percentile was positively associated with memory (r = 0.36)
Scott et al. (2016) [20]	Cross-sectional	120 healthy women aged 18–35 years	Indirect calorimetry; max test	Working memory	Not mentioned	No	Positive association between CRF and working memory (accuracy) (β = 0.15, *p* = 0.006)
Hayes et al. (2016) [21]	Cross-sectional	*n* = 33 Young adults (age 18–31 years)and *n* = 28 older adults (age 55–82 years); sample mean V˙O_2peak_ of 44.6 mL·min^–1^·kg^–1^	Indirect calorimetry; max test	Episodic memory	Not mentioned	No	CRF was not associated with memory in young adults (F < 1), but was positively associated with memory in older adults (F = 5.40, *p* < 0.05)
Dougherty et al. (2017) [28]	Cross-sectional	*n* = 1500; 86 cognitive healthy, M_age_ = 63.6 years, 57 analytic sample, M_age_ = 62.6 years, 65 for CRF and episodic memory analysis, M_age_ = 62.9 years	Indirect calorimetry; max test	Episodic memory (RAVLT)	Not mentioned. But participants were not enrolled in any exercise trials at the start of the study	No	No overall significant association between CRF and memory, but CRF was positively and significantly associated with memory among men; delayed recall score (*R*^2^ = 0.23, *p* = 0.026) and composite memory score (*R*^2^ = 0.19, *p* = 0.049)
Hayes et al. (2017) [23]	Cross-sectional	*n* = 26 Older Adults (55–74 years), 31 Younger Adults (18–31 years) M_age_ = 29.3 and 63.6 years; VO_2max_ = 25.9 mL/kg/min	Indirect calorimetry; max test	Source memory (face-name recognition)	Not mentioned	No	Peak CRF accounted for 43.7% of the variance in source memory (F = 18.64, *p* < 0.001)
Hwang et al. (2017) [22]	Cross-sectional	*n* = 87 young adults (18–29); M_age_ = 23.22; 57% female; 43% male; VO2_max_ = 37.78 mL/kg/min	Indirect calorimetry; max test	Working memory	Not mentioned	No	Higher CRF was associated with higher correct trials (F = 4.70, *p* = 0.03) and shorter memory retrieval latency (F = 7.00, *p* < 0.01)
Schwarb et al. (2017) [27]	Cross-sectional	*n* = 63; M_age_ = 22.9 y; Relative VO_2max_ = 42.1 mL/kg/min	Indirect calorimetry; max test	Relational memory	Not mentioned.	No	CRF was positively associated with relationship memory (r = 0.29, *p* = 0.04).
Brown et al. (2019) [26]	Cross-sectional	*n* = 99 aged 60–80; M_age_ = 69 years; VO_2peak =_ 23 mL kg^−1^ min^−1;^ M_age_ = 67 years for higher fit group; M_age_ = 71.5 years for lower fit group	Indirect calorimetry; max test	Verbal, visuospatial memory, and paired associative memory	Not mentioned.	No	No difference between the lower-fit and higher-fit groups were observed on the CVALT delayed recall (F = 0.73, *p* = 0.40) and BVMT delayed recall (F = 0.05, *p* = 0.83). However, the higher-fit group performed better than the lower-fit group (in terms of errors) on the paired associative task (F = 7.94, *p* = 0.006).
